# Mechanisms of main components in *Curcuma longa L.* on hepatic fibrosis based on network pharmacology and molecular docking: A review

**DOI:** 10.1097/MD.0000000000034353

**Published:** 2023-07-21

**Authors:** Qiang Han, Jiahui Zhu, Peng Zhang

**Affiliations:** a Department of Traditional Chinese Medicine, Health Service Center of Beiyuan Community, Beijing, China; b Faculty of Rehabilitation Medicine, Binzhou Medical University, Yantai, Shandong Province, China; c Department of Gastroenterology and Hepatology, Beijing University of Chinese Medicine Affiliated Dongfang Hospital, Beijing, China.

**Keywords:** *Curcuma longa L.*, hepatic fibrosis, molecular docking, network pharmacology

## Abstract

Hepatic fibrosis is a great concern in public health. While effective drugs for its treatment are lacking, *Curcuma longa L.* (CL) has been reported as a promising therapeutic. We aimed to uncover the core components and mechanisms of CL against hepatic fibrosis via a network pharmacology approach.

**Methods:**

The main components of CL were obtained and screened. While targets of components and disease were respectively collected using SwissTargetPrediction and online databases, common targets were assessed. A protein–protein interaction (PPI) network was constructed, and core targets were identified. GO and KEGG pathway enrichment analyses were performed, and molecular docking was conducted to validate the binding of core components in CL on predicted core targets.

**Results:**

Nine main components from CL based on high-performance liquid chromatography (HPLC) and 63 anti-fibrosis targets were identified, and a PPI network and a component target-disease target network were constructed. Apigenin, quercetin, demethoxycurcumin, and curcumin are likely to become key phenolic-based components and curcuminoids for the treatment of hepatic fibrosis, respectively. KEGG pathway enrichment analysis revealed that the HIF-1 signaling pathway (hsa04066) was most significantly enriched. Considering core targets of the PPI network and a network of the common targets and pathways enriched, AKT1, MAPK1, EGFR, MTOR, and SRC may be the core potential targets of CL against hepatic fibrosis. Molecular docking was carried out to verify the binding of above core components to core targets.

**Conclusions:**

The therapeutic effect of CL on hepatic fibrosis may be attributed to multi-components, multi-targets, and multi-pathways.

## 1. Introduction

Hepatic fibrosis is a reversible wound-healing response characterized by the accumulation of extracellular matrix (ECM) following liver injury by, for example, hepatitis B virus and/or hepatitis C virus infections, alcohol, or metabolic syndrome inducing nonalcoholic steatohepatitis. Perpetuation of hepatic fibrosis results in cirrhosis, which is the 16th leading cause of death and severely affects the quality of life.^[1]^ Efforts to explore medicines facilitating fibrosis resolution will reduce the incidence of end stage liver diseases. Studies have revealed key mechanisms leading to liver fibrosis, which include chronic damage to hepatocytes and the epithelial or endothelial barrier, release of inflammatory cytokines, recruitment of inflammatory cells, activation of hepatic myofibroblasts, excessive production of ECM and dysregulation of ECM degradation.^[2,3]^ However, over the last decade, despite a variety of experimental and clinical studies targeting single mechanism, there is still no effective drug to treat hepatic fibrosis in the clinic. Etiology-focused therapies are the main treatment for patients, which cannot directly contribute to fibrosis resolution and liver repair. Given that multiple pathologies are associated with hepatic fibrosis, the development of novel therapeutics targeting multiple pathogenic pathways is desirable.

Traditional Chinese medicine (TCM) has been used to treat hepatic fibrosis for thousands of years, and both bioactive components and formulae have been proved to be effective.^[4,5]^ *Curcuma longa L.* (CL), known as “Jianghuang” in Chinese, is widely used as a food spice and herbal medicine in Asia. Classical TCM documents, such as Tang Materia Medica and Rihuazi Materia Medica, demonstrated that CL can promote blood circulation and remove stasis, and treat abdominal mass, etc. Clinically, formulae containing CL, such as Shengjiang Powder, Zhongman Fenxiao Pill, and Jianghuang Powder, have also been widely used to treat chronic liver diseases. Researches have demonstrated the therapeutic effects of CL on carbon tetrachloride induced liver injury, liver fibrosis, and cirrhosis, suggesting that CL has the potential as a treatment option for hepatic fibrosis.^[6,7]^ The effects of CL are largely attributed to its predominant active components. Previous studies showed that CL consists of different components with following percentage; curcuminoid compounds 2% to 5%, carbohydrates nearly 40% to 70%, proteins 6% to 8%, oils 5% to 8%, and minerals and other elements 3% to 5%. Curcuminoids and essential oils are 2 major components present in CL.^[8–10]^ Salama et al showed Hepatoprotective effect of ethanolic extract of CL on thioacetamide induced liver cirrhosis in rats.^[11]^ Lin et al found that curcumin, the main compound of CL, exerted anti-fibrogenic effects and induction of apoptosis in rat hepatic stellate cells (HSCs).^[12]^ Remarkable hepatoprotective, anti-inflammatory, and anti-fibrotic effects of analogs of curcumin and non-curcuminoid constituents have also been reported.^[13,14]^ Despite a series of studies on the biological activities of active components in CL, the potential targets and underlying mechanisms of CL in hepatic fibrosis remain largely unclear. Due to the effects of CL and its bioactives on hepatic fibrosis in researches and a variety of pathological processes involved, we therefore hypothesized that CL possibly exert a treatment effect by acting on multiple pathological processes.

**Figure 1.**
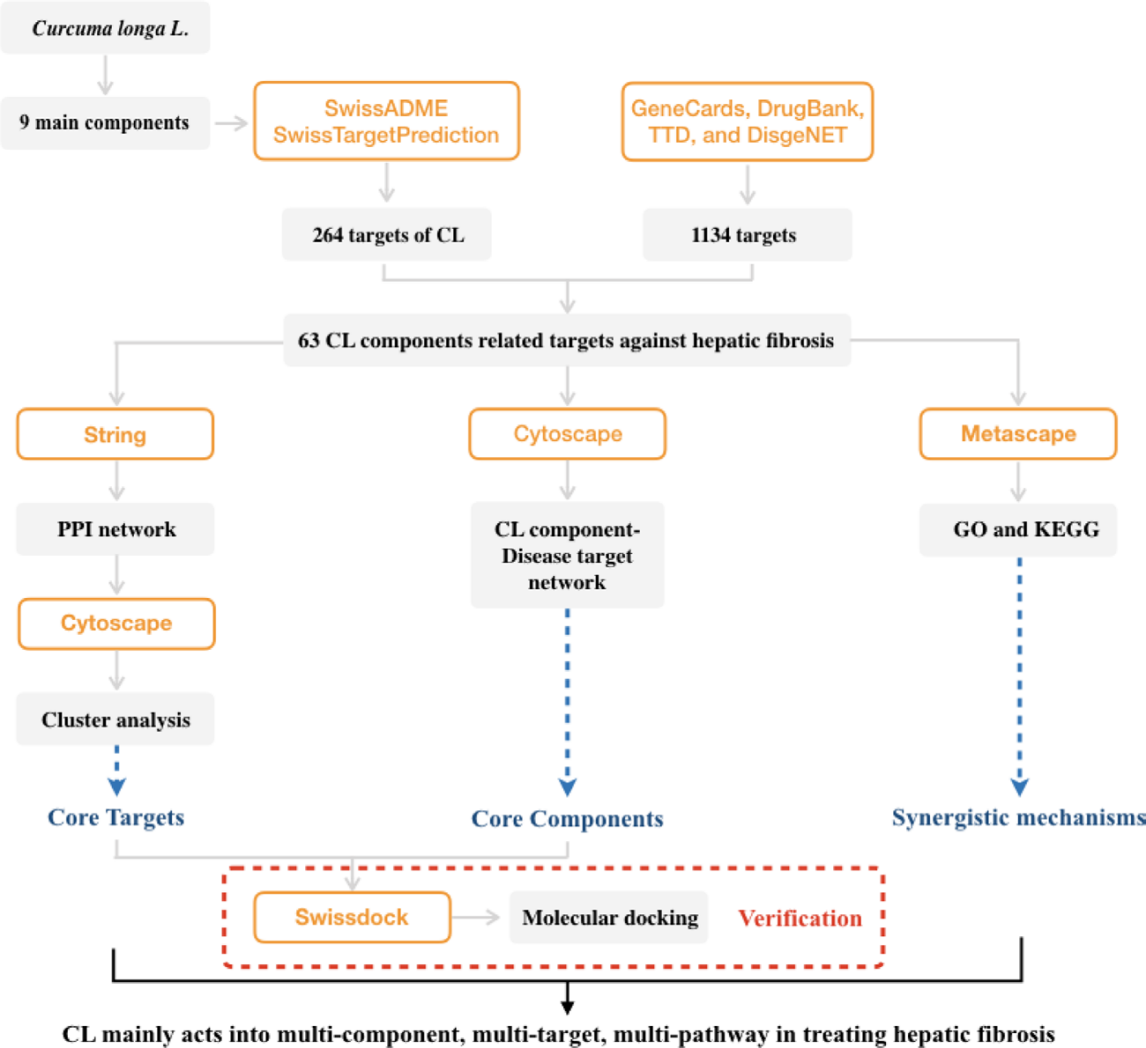
Flowchart of the study.

As an approach for drug discovery, network pharmacology combines network biology and multi-pharmacology. This study aimed to identify effective target proteins of main components in CL and uncover the mechanism of the anti-fibrosis effect through a network pharmacology approach and molecular docking. A scheme of the study protocol is shown in Figure 1. We obtained main components of CL from a recent study based on high-performance liquid chromatography (HPLC),^[15]^ which increased the quality of this study significantly. Our study may offer new insights into the mechanisms of CL and provides a more specific and effective treatment for hepatic fibrosis.

## 2. Methods

### 2.1. Screening of main active components in CL and related targets

The main components in CL were obtained from a recent study based on HPLC.^[13]^ the CAS numbers of the relevant molecules were obtained. The chemical structures were found through the PubChem website and saved as sdf files, which then were used to predict the oral availability and drug-like properties of the components on the SwissADME website (http://www.swissadme.ch [accessed on February 10, 2022]).^[16]^ The screening condition was “GIabsorption” as “High,” and “Druglikeness” (Lipinski, Ghose, Veber, Egan, Muegge) met 3 of the 5 rules. This finally determined the main active components of CL. To obtain the targets of the main active components, SwissTargetPrediction (http://www.swisstargetprediction.ch/ [accessed on February 10, 2022])^[17]^ was employed.

### 2.2. Screening of common targets of the main components against hepatic fibrosis

Disease targets were collected from the GeneCards database (https://auth.lifemapsc.com/now [accessed on February 10, 2022]), DrugBank database (https://go.drugbank.com/ [accessed on February 10, 2022]),^[18]^ therapeutic target Database (https://db.idrblab.net/ttd/ now. [accessed on February 10, 2022]),^[19]^ and DisgeNET database (https://www.disgenet.org/ [accessed on February 10, 2022]) using “hepatic fibrosis,” “liver fibrosis,” or “cirrhosis” as a key phrase, and duplicate targets were removed using Microsoft Excel software. The intersection of CL-related targets and disease targets was assessed by Venny 2.1 (https://bioinfogp.cnb.csic.es/tools/venny/index.html [accessed on February 10, 2022]). Common targets represent the target of main active components in CL against hepatic fibrosis.

### 2.3. Protein–protein interaction (PPI) network construction and clustering analysis

A PPI network was constructed by using the STRING database version 11.5 (https://string-db.org/ [accessed on February 10, 2022]).^[20]^ The organism was set to *Homo sapiens*, and only the minimum required interaction score > 0.4 was chosen as significant. PPI networks consist of nodes, which represent a target protein, and edges, which represent PPI. The thickness of an edge represents the combined score. Degree refers to the number of other nodes directly connected to a node. The higher the degree is, the more important the node is. Core targets were identified through network analysis using Cytoscape software (v.3.9.1)^[21]^ and its plugin (Network Analysis). In the present study, the top 10 proteins ranked by degree were selected and identified as core targets.

The Cytoscape plugin Molecular Complex Detection (MCODE)^[22]^ was applied to analyze clustering modules in the PPI network. The MCODE criteria for selection were as follows: degree cutoff = 2, node score cutoff = 0.2, k-core = 2, and max depth = 100. A node with the highest weighted vertex was defined as a seed node, the key target of this cluster, by MCODE. Moreover, a potential CL target-hepatic fibrosis target network was constructed using Cytoscape software.

### 2.4. GO and KEGG pathway enrichment analyses

Metascape (https://metascape.org/gp [accessed on February 10, 2022])^[23]^ is a comprehensive tool for gene annotation and enrichment analysis. GO and KEGG pathway enrichment analyses were performed using Metascape. The enriched terms with *P* < .01, a minimum count of 3, and an enrichment factor >1.5 were considered significant. The top 20 enriched terms were visualized using an online tool (www.bioinformatics.com.cn [accessed on February 10, 2022]). A target-enriched KEGG pathway network for the main components against hepatic fibrosis was also constructed by Cytoscape software. Red nodes represent enriched KEGG pathways, and yellow nodes represent target proteins.

### 2.5. Molecular docking

To validate the binding of core components in CL on predicted core targets, the 3D molecular structure of compounds was retrieved from the PubChem database and the structure files of target proteins were acquired from the RCSB Protein Data Bank (database, http://www.rcsb.org/ [accessed on February 10, 2022]).^[24]^ Molecular docking calculations were performed using the SwissDock web service (http://www.swissdock.ch/docking [accessed on February 10, 2022]).^[25]^

## 3. Results

### 3.1. The chemical structure and ADME properties of the main components from CL

**Figure 2.**
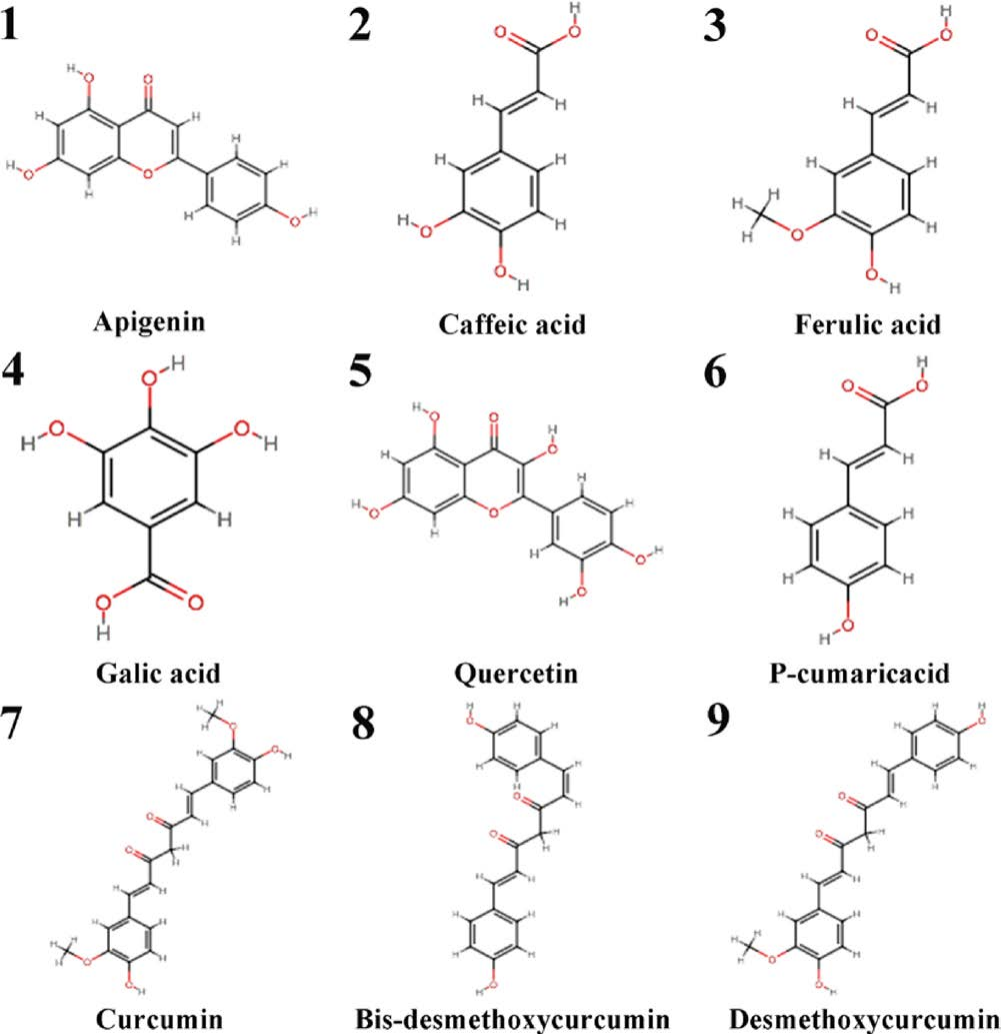
Structures of the main components extracted from *Curcuma longa L.* (CL).

Based on the HPLC results from a recent study, the main phenolic-based components and curcuminoids in CL were reported as follow, rutin, ferulic acid, caffeic acid, apigenin, p-cumaric acid, quercetin, gallic acid, curcumin, bis-desmethoxycurcumin, and desmethoxycurcumin. The chemical structures of the main components were obtained from the PubChem database and shown in Figure 2. Then, we evaluated the ADME-related properties of the 10 components via an online tool SwissADME. Most components satisfied the screening condition, except for rutin (Table 1), which means that these components may exhibit good permeability across cell membranes.

**Table 1:** Pharmacological and molecular properties of the main components in CL.

Name	Formula	MW (g/mol)	Hdon	Hacc	Rbon	TPSA(Å²)	LogP	LogS	Log Kp (cm/s)
ferulic acid	C10H10O4	194.18	2	4	3	66.76	1.36	−2.52	−6.41
caffeic acid	C9H8O4	180.16	3	4	2	77.76	0.93	−2.38	−6.58
apigenin	C15H10O5	270.24	3	5	1	90.90	2.11	−4.59	−5.80
*p*-cumaric acid	C9H8O3	164.16	2	3	2	57.53	1.26	−2.27	−6.26
quercetin	C15H10O7	302.24	5	7	1	131.36	1.23	−3.91	7.05
gallic acid	C7H6O5	170.12	4	5	1	97.99	0.21	−2.34	−6.84
curcumin	C21H20O6	368.38	2	6	8	93.06	3.03	−4.83	−6.28
bis-desmethoxycurcumin	C19H16O4	308.33	2	4	6	74.60	2.83	−4.50	−5.87
desmethoxycurcumin	C20H18O5	338.35	2	5	7	83.83	3.00	−4.76	−6.01

CL = *Curcuma longa* L., Hacc = hydrogen bond acceptors, Hdon = hydrogen bond donors, Log Kp = skin permeation, LogP = lipid–water partition coefficient, LogS = solubility, MW = molecule weight, Rbon = rotatable bonds, TPSA = topological polar surface area.

### 3.2. Screening targets of the main components from CL in hepatic fibrosis

**Figure 3.**
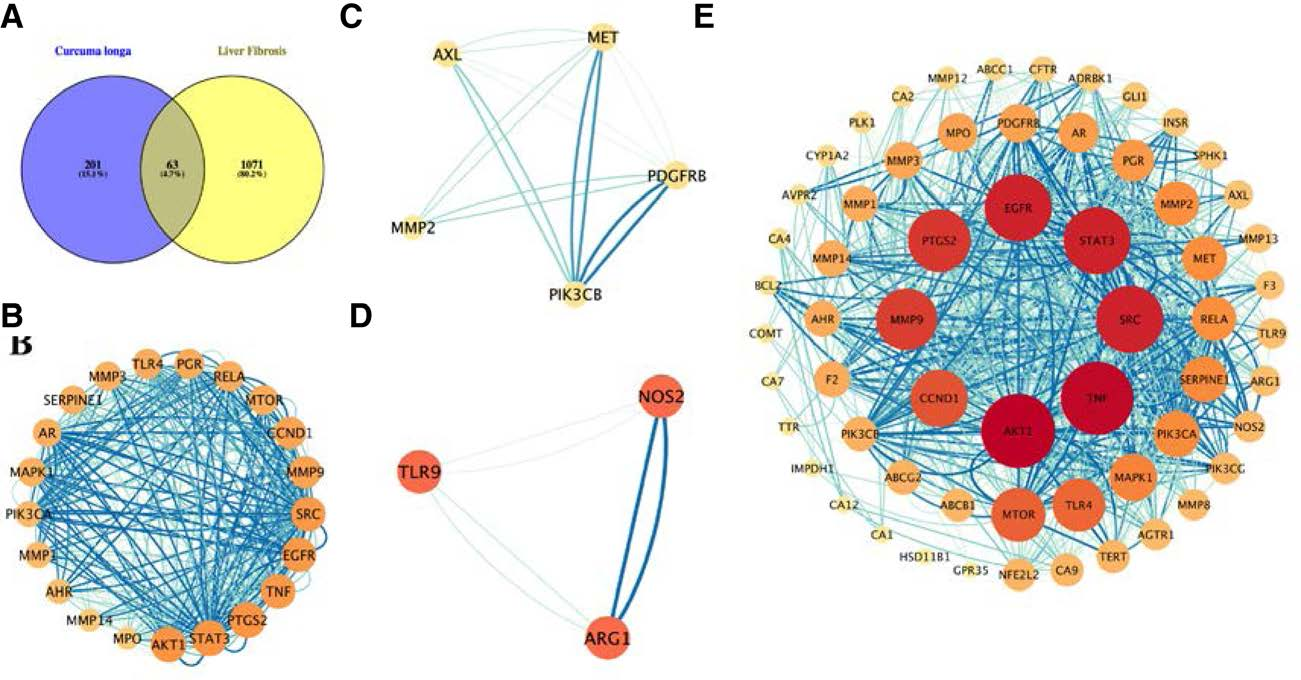
The protein–protein interaction (PPI) network and clusters of common targets of CL components against hepatic fibrosis. (A) A Venn diagram was applied to obtain the intersection between the components in CL and targets of hepatic fibrosis. (B–D) Clusters 1 to 3 were found with Molecular Complex Detection (MCODE), which can identify densely connected regions. (E) PPI network of CL components against hepatic fibrosis. Nodes represent target proteins, and edges represent interactions among targets. The darker the color and the larger the node are, the greater the degree is. CL = Curcuma longa L.

Potential targets of the components were predicted by using the SwissTargetPrediction database based on their structure, and a total of 264 targets were obtained. Results from the GeneCards, DrugBank, Therapeutic Target Database, and DisgeNET identified a total of 1134 targets relevant to hepatic fibrosis. A Venn diagram was used to summarize 63 common targets associated with both core components of CL and hepatic fibrosis for further analysis (Fig. 3A). Detailed information about these common targets is provided in Table 2.

**Table 2:** Targets of CL components against hepatic fibrosis.

Number	Gene ID	Gene symbol	Description
1	760	CA2	Carbonic anhydrase 2
2	766	CA7	Carbonic anhydrase 7
3	759	CA1	Carbonic anhydrase 1
4	771	CA12	Carbonic anhydrase 12
5	768	CA9	Carbonic anhydrase 9
6	4318	MMP9	Matrix metalloproteinase-9
7	4312	MMP1	Interstitial collagenase
8	4313	MMP2	72 kDa type IV collagenase
9	4780	NFE2L2	Nuclear factor erythroid 2-related factor 2
10	6774	STAT3	Signal transducer and activator of transcription 3
11	3290	HSD11B1	Corticosteroid 11-beta-dehydrogenase isozyme 1
12	762	CA4	Carbonic anhydrase 4
13	7099	TLR4	Toll-like receptor 4
14	4233	MET	Hepatocyte growth factor receptor
15	1544	CYP1A2	Cytochrome P450 1A2
16	1956	EGFR	Epidermal growth factor receptor
17	5743	PTGS2	Prostaglandin G/H synthase 2
18	7276	TTR	Transthyretin
19	5970	RELA	Transcription factor p65
20	54106	TLR9	Toll-like receptor 9
21	57016	AKR1B10	Aldo-keto reductase family 1 member B10
22	2152	F3	Tissue factor
23	4843	NOS2	Nitric oxide synthase, inducible
24	595	CCND1	G1/S-specific cyclin-D1
25	5243	ABCB1	ATP-dependent translocase ABCB1
26	196	AHR	Aryl hydrocarbon receptor
27	5594	MAPK1	Mitogen-activated protein kinase 1
28	5291	PIK3CB	Phosphatidylinositol 4,5-bisphosphate 3-kinase catalytic subunit beta isoform
29	5290	PIK3CA	Phosphatidylinositol 4,5-bisphosphate 3-kinase catalytic subunit alpha isoform
30	4363	ABCC1	Multidrug resistance-associated protein 1
31	1080	CFTR	Cystic fibrosis transmembrane conductance regulator
32	9429	ABCG2	Broad substrate specificity ATP-binding cassette transporter ABCG2
33	4321	MMP12	Macrophage metalloelastase
34	383	ARG1	Arginase-1
35	367	AR	Androgen receptor
36	7015	TERT	Telomerase reverse transcriptase
37	5347	PLK1	Serine/threonine-protein kinase PLK1
38	558	AXL	Tyrosine-protein kinase receptor UFO
39	2859	GPR35	G-protein coupled receptor 35
40	2147	F2	Prothrombin
41	554	AVPR2	Vasopressin V2 receptor
42	4353	MPO	Myeloperoxidase
43	5836	PYGL	Glycogen phosphorylase, liver form
44	6714	SRC	Proto-oncogene tyrosine-protein kinase Src
45	4322	MMP13	Collagenase 3
46	4314	MMP3	Stromelysin-1
47	207	AKT1	RAC-alpha serine/threonine-protein kinase
48	5241	PGR	Progesterone receptor
49	3643	INSR	Insulin receptor
50	5294	PIK3CG	Phosphatidylinositol 4,5-bisphosphate 3-kinase catalytic subunit gamma isoform
51	5054	SERPINE1	Plasminogen activator inhibitor 1
52	1312	COMT	Catechol O-methyltransferase
53	4323	MMP14	Matrix metalloproteinase-14
54	185	AGTR1	Type-1 angiotensin II receptor
55	4317	MMP8	Neutrophil collagenase
56	3614	IMPDH1	Inosine-5’-monophosphate dehydrogenase 1
57	8877	SPHK1	Sphingosine kinase 1
58	596	BCL2	Apoptosis regulator Bcl-2
59	5159	PDGFRB	Platelet-derived growth factor receptor beta
60	156	GRK2	Beta-adrenergic receptor kinase 1
61	2735	GLI1	Zinc finger protein GLI1
62	2475	MTOR	Serine/threonine-protein kinase mTOR
63	7124	TNF	Tumor necrosis factor

CL = *Curcuma longa* L.

### 3.3. PPI analysis of targets of the main components against hepatic fibrosis

To explore the therapeutic mechanism of the main components in the treatment of hepatic fibrosis, the PPI analysis was performed using the STRING database and visualized by Cytoscape software (v.3.9.1) (Fig. 3E). A PPI network with a total of 63 nodes and 450 edges and an average node degree of 14.3 was generated. The darker the color and the larger the node were, the greater the degree was. The darker the color and the wider the edge were, the greater the combined score was. AKT1, TNF, STAT3, EGFR, SRC, PTGS2, MMP9, CCND1, MTOR, and TLR4, which were ranked by degree, were identified as core targets. Among these, AKT1 was shown with the highest degree. It means that these core targets are closely related to other targets in the PPI network, and these core targets may play important roles in the treatment of hepatic fibrosis.

### 3.4. Clusters of common targets of the main components against hepatic fibrosis

Three clusters were found in the PPI network through MCODE (k-core = 2), which may be the most relevant to AD treatment. The details are provided in Figure 3B to D. Cluster 1 contains 21 nodes and 320 edges with a score of 16. The seed node of this cluster is MAPK1 which encodes mitogen-activated protein kinase 1 that mediates diverse biological functions such as cell growth, adhesion, survival and differentiation through the regulation of transcription, translation, cytoskeletal rearrangements. Cluster 2 contains 5 nodes and 16 edges with a score of 4. The seed node of this cluster is MET, which encodes hepatocyte growth factor receptor that transduces signals from the ECM into the cytoplasm by binding to hepatocyte growth factor ligand. Cluster 3 contains 3 nodes and 6 edges with a score of 3. The seed node of this cluster is ARG1, which encodes Arginase-1 and contributes to collagen synthesis and bioenergetic pathways critical for cell proliferation.

### 3.5. Construction of a main component target-disease target network

**Figure 4.**
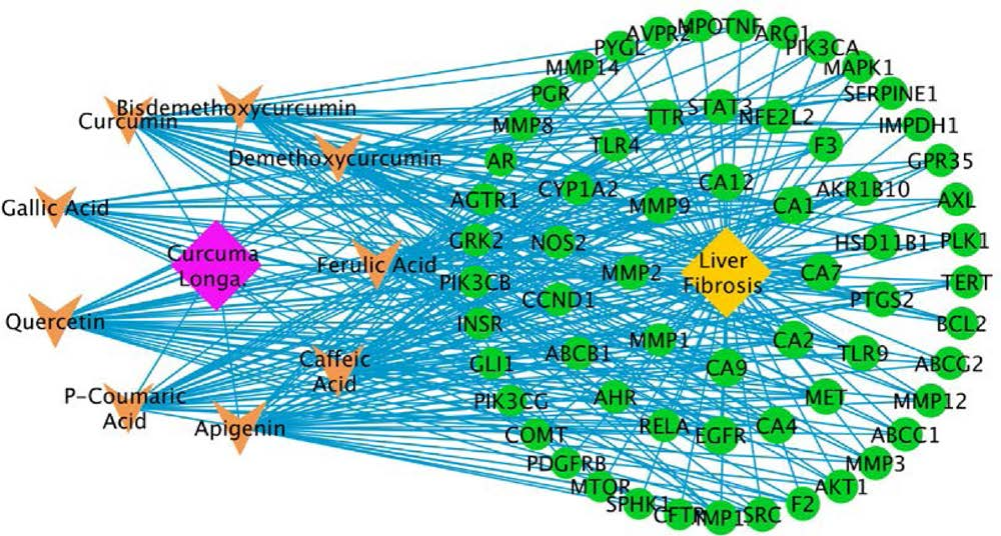
The main component target-disease target network. Orange nodes represent the main components extracted from CL. Green nodes represent common targets between potential targets of CL components and targets of hepatic fibrosis. CL = *Curcuma longa L.*

63 common targets and 9 main active components from CL were used to construct a main component target-disease target network (Fig. 4). All components were associated with multiple targets, resulting in 310 component-target associations. The average number of targets per component was 25.6, and the mean degree of components per target was 4.78, which indicates that CL fits the multi-component and multi-target characteristics of TCM. Apigenin (degree = 34) had the most targets, followed by quercetin (degree = 33), caffeic acid (31), ferulic acid (30), p-coumaric acid (28), demethoxycurcumin (27), curcumin (degree = 26), bisdemethoxycurcumin (21), and gallic acid (17), suggesting that these components may be the core components in the treatment of hepatic fibrosis.

### 3.6. GO and KEGG pathway enrichment analysis

**Figure 5.**
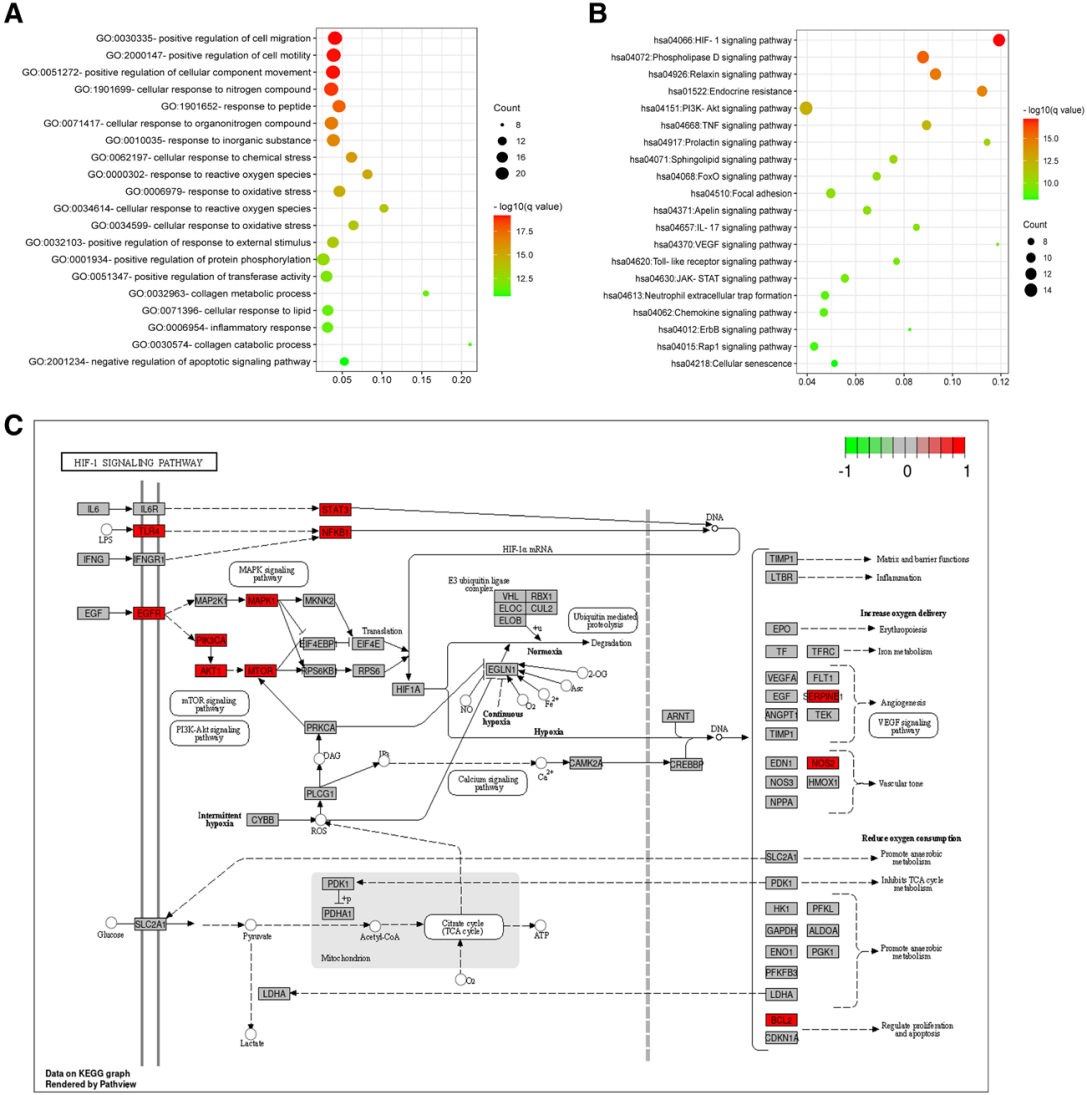
GO biological process (A) terms and the results of KEGG (B) pathway enrichment analysis of target proteins of main components in CL against hepatic fibrosis. The x-axis represents the rich factor, the bubble size represents the number of targets enriched in terms, and the color indicates the *q*-value. (C) Schematic drawing of the HIF-1 signaling pathway (hsa04066). CL = *Curcuma longa L*.

The GO and KEGG pathway enrichment analysis of 63 common targets were performed relying on Metascape platform according to the *P* < .01. There were 902, 59, and 80 GO terms related to biological processes (BP), cell components, and molecular functions (MF), respectively. The primary enriched BP terms were positive regulation of cell migration (GO:0030335), positive regulation of cell motility (GO:2000147), positive regulation of cellular component movement (GO:0051272), etc (Fig. 5A). For cell components, the targets were enriched in ECM (GO:0031012), external encapsulating structure (GO:0030312), vesicle lumen (GO:0031983), etc. MF analysis revealed including carbonate dehydratase activity (GO:0004089), phosphotransferase activity, alcohol group as acceptor (GO:0016773), kinase activity (GO:0016301), etc.

**Figure 6.**
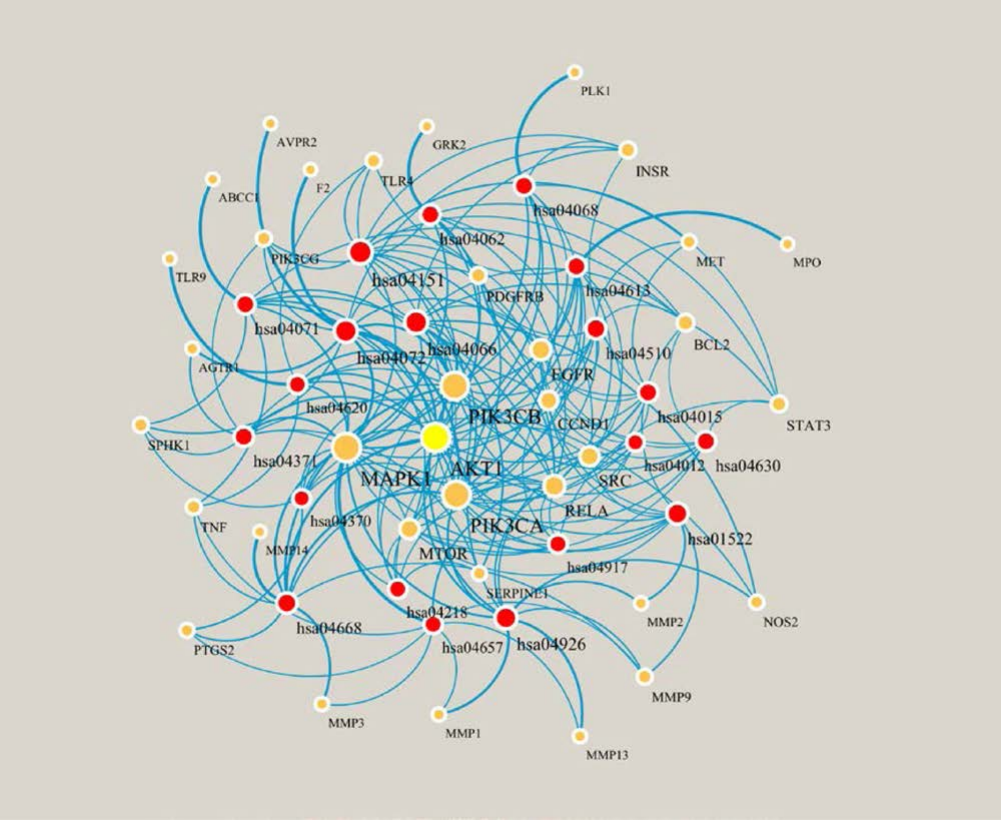
Target-enriched KEGG pathway network for CL components against hepatic fibrosis. Red nodes represent enriched KEGG pathways, and yellow nodes represent target proteins. The darker the color and the larger the node are, the greater the degree is.

In addition, 148 pathways were contained in KEGG pathway enrichment analysis, mainly including HIF-1 signaling pathway (hsa04066), Phospholipase D signaling pathway (hsa04072), Relaxin signaling pathway (hsa04926), Endocrine resistance (hsa01522), and PI3K-Akt signaling pathway (hsa04151) (Fig. 5B). Detailed information on the KEGG pathway enrichment analysis is shown in Table 3. The most significantly enriched pathway, HIF-1 signaling pathway (hsa04066, *q* = 6.49E-18), plays an important role in the adaptation to hypoxic conditions and is closely related to vascularization, metabolic regulation, cell multiplication and survival (Fig. 5C). A network of the targets and pathways enriched in the core components against hepatic fibrosis is shown in Figure 6 and contains 55 nodes, including the top 20 KEGG pathways associated with 35 targets and 192 edges. The top 10 degree values were AKT1, MAPK1, PIK3CB, PIK3CA, RELA, EGFR, MTOR, SRC, CCND1, and BCL2. Together with mapping of the PPI network and main enriched pathways, it is speculated that AKT1, MAPK1, EGFR, MTOR, and SRC may be the key targets of CL.

**Table 3:** Top 20 KEGG pathway terms enriched in CL components against hepatic fibrosis.

Term	Pathway	Rich factor	q-Value	Count	Symbols
hsa04066	HIF-1 signaling pathway	0.12	6.491E-18	13	AKT1, BCL2, EGFR, MTOR, INSR, NOS2, SERPINE1, PIK3CA, PIK3CB, MAPK1, RELA, STAT3, TLR4
hsa04072	Phospholipase D signaling pathway	0.09	2.279E-16	13	AGTR1, AKT1, AVPR2, EGFR, F2, MTOR, INSR, PDGFRB, PIK3CA, PIK3CB, PIK3CG, MAPK1, SPHK1
hsa04926	Relaxin signaling pathway	0.09	1.661E-15	12	AKT1, EGFR, MMP1, MMP2, MMP9, MMP13, NOS2, PIK3CA, PIK3CB, MAPK1, RELA, SRC
hsa01522	Endocrine resistance	0.11	3.930E-15	11	AKT1, CCND1, BCL2, EGFR, MTOR, MMP2, MMP9, PIK3CA, PIK3CB, MAPK1, SRC
hsa04151	PI3K-Akt signaling pathway	0.04	3.232E-13	14	AKT1, CCND1, BCL2, EGFR, MTOR, INSR, MET, PDGFRB, PIK3CA, PIK3CB, PIK3CG, MAPK1, RELA, TLR4
hsa04668	TNF signaling pathway	0.09	6.552E-13	10	AKT1, MMP3, MMP9, MMP14, PIK3CA, PIK3CB, MAPK1, PTGS2, RELA, TNF
hsa04917	Prolactin signaling pathway	0.11	2.134E-11	8	AKT1, CCND1, PIK3CA, PIK3CB, MAPK1, RELA, SRC, STAT3
hsa04071	Sphingolipid signaling pathway	0.08	3.169E-11	9	AKT1, BCL2, ABCC1, PIK3CA, PIK3CB, MAPK1, RELA, TNF, SPHK1
hsa04068	FoxO signaling pathway	0.07	6.683E-11	9	AKT1, CCND1, EGFR, INSR, PIK3CA, PIK3CB, PLK1, MAPK1, STAT3
hsa04510	Focal adhesion	0.05	1.021E-10	10	AKT1, CCND1, BCL2, EGFR, MET, PDGFRB, PIK3CA, PIK3CB, MAPK1, SRC
hsa04371	Apelin signaling pathway	0.06	1.042E-10	9	AGTR1, AKT1, CCND1, MTOR, NOS2, SERPINE1, PIK3CG, MAPK1, SPHK1
hsa04657	IL-17 signaling pathway	0.09	1.596E-10	8	MMP1, MMP3, MMP9, MMP13, MAPK1, PTGS2, RELA, TNF
hsa04370	VEGF signaling pathway	0.12	2.730E-10	7	AKT1, PIK3CA, PIK3CB, MAPK1, PTGS2, SRC, SPHK1
hsa04620	Toll-like receptor signaling pathway	0.08	3.349E-10	8	AKT1, PIK3CA, PIK3CB, MAPK1, RELA, TLR4, TNF, TLR9
hsa04630	JAK-STAT signaling pathway	0.06	3.581E-10	9	AKT1, CCND1, BCL2, EGFR, MTOR, PDGFRB, PIK3CA, PIK3CB, STAT3
hsa04613	Neutrophil extracellular trap formation	0.05	1.432E-09	9	AKT1, MTOR, MPO, PIK3CA, PIK3CB, MAPK1, RELA, SRC, TLR4
hsa04062	Chemokine signaling pathway	0.05	1.543E-09	9	GRK2, AKT1, PIK3CA, PIK3CB, PIK3CG, MAPK1, RELA, SRC, STAT3
hsa04012	ErbB signaling pathway	0.08	3.133E-09	7	AKT1, EGFR, MTOR, PIK3CA, PIK3CB, MAPK1, SRC
hsa04015	Rap1 signaling pathway	0.04	3.181E-09	9	AKT1, EGFR, INSR, MET, PDGFRB, PIK3CA, PIK3CB, MAPK1, SRC
hsa04218	Cellular senescence	0.05	7.013E-09	8	AKT1, CCND1, MTOR, SERPINE1, PIK3CA, PIK3CB, MAPK1, RELA

CL = Curcuma longa L.

### 3.7. Molecular docking simulation

From the main component target-disease target network (Fig. 5), apigenin (degree = 34), quercetin (degree = 33), demethoxycurcumin (degree = 27), curcumin (degree = 26), had the highest number of targets against hepatic fibrosis in phenolic-based components and curcuminoids, respectively. Therefore, molecular docking analysis was used to validate the binding of above main components to speculated key targets, namely AKT1, MAPK1, EGFR, MTOR, and SRC. Delta G is defined as the binding energy based on the ensemble free energy; the greater the absolute value of Delta G is, the more stable binding is. The results of molecular docking are shown in Table 4.

**Table 4:** Molecular docking of core targets with potential core components.

Ligands	Target	PDB	deltaG (kcal/mol)	FullFitness (kcal/mol)
apigenin	AKT1	7NH5	−7.15	−2186.99
apigenin	MAPK1	6GJD	−7.79	−1926.14
apigenin	EGFR	5CNN	−7.26	−1968.04
apigenin	MTOR	5WBH	−7.09	−3766.17
apigenin	SRC	7NG7	−6.98	−1702.68
quercetin	AKT1	7NH5	−7.15	−2158.85
quercetin	MAPK1	6GJD	−7.91	−1907.44
quercetin	EGFR	5CNN	−8.08	−1942.2
quercetin	MTOR	5WBH	−7.63	−3740.89
quercetin	SRC	7NG7	−8.06	−1678
demethoxycurcumin	AKT1	7NH5	−8.06	−2177.25
demethoxycurcumin	MAPK1	6GJD	−9.5	−1922.55
demethoxycurcumin	EGFR	5CNN	−8.98	−1955.34
demethoxycurcumin	MTOR	5WBH	−8.03	−3754.66
demethoxycurcumin	SRC	7NG7	−8.65	−1699.02
curcumin	AKT1	7NH5	−8.3	−2173.97
curcumin	MAPK1	6GJD	−8.9	−1921.45
curcumin	EGFR	5CNN	−8.85	−1960.27
curcumin	MTOR	5WBH	−8.25	−3752.11
curcumin	SRC	7NG7	−8.43	−1701.93

PDB = protein data bank.

## 4. Discussion

Many in vitro studies have reported the hepatoprotective, antioxidant, antisteatotic and antilipidemic, anti-inflammatory, anti-fibrotic, antitumor, and cholagogic effects of the main phenolic-based components and curcuminoids in CL.^[11–14,26,27]^ Unfortunately, the potential targets and mechanisms of CL are still not clear at present. This study comprehensively investigated the therapeutic effect of main components of CL on hepatic fibrosis via network pharmacology.

Generally, network pharmacology studies use public databases to obtain the main components in TCM. Conventional screening methods of public databases do not take into account the content and distribution specificity of components. In our study, the main phenolic-based components and curcuminoids in CL were obtained by HPLC based on a recent study,^[15]^ which greatly improved the quality of the data collected. Totally, 10 components were included, and 9 of 10 were confirmed as potential core active components. 63 common targets of these 9 components against hepatic fibrosis were obtained using network pharmacology strategies. According to the PPI analysis of 63 co-targets, AKT1, TNF, STAT3, EGFR, SRC, PTGS2, MMP9, CCND1, MTOR, and TLR4, which have the highest degrees, may be the main targets of CL against hepatic fibrosis. MAPK1, MET, and ARG1 were also found as seed nodes of 3 clusters in PPI network via MCODE. Potential core targets will be discussed later in combination with the results of KEGG analysis and molecular docking.

A main component target-disease target network indicated that CL may act in a multi-component and multi-target way. Apigenin, quercetin, demethoxycurcumin, and curcumin are likely to become key phenolic-based components and curcuminoids for the treatment of hepatic fibrosis, respectively. Apigenin, a natural potent antioxidant, has the largest number of therapeutic targets for hepatic fibrosis (degree = 34). Studies showed that apigenin can alleviate hepatic fibrosis by inhibiting HSC activation and autophagy via TGF-β1/Smad3 and p38/PPARα pathways, and regulating VEGF-mediated FAK phosphorylation through the MAPKs, PI3K/Akt, HIF-1, ROS, and eNOS pathways.^[28–30]^ Quercetin is the major representative of the flavonoid subclass of flavones, widely found in fruits, vegetables, and many herbal medicines. Experiments demonstrated that quercetin might inhibit liver inflammation through regulating NF-κB/TLR/ NLRP3 and reducing PI3K/Nrf2-mediated oxidative stress, and improve hepatic fibrosis by inhibiting HSC activation and regulating pro-fibrogenic/anti-fibrogenic molecules balance.^[31–33]^ Curcumin, the principal curcuminoid of CL, has been reported to show antitumor, antioxidant, and anti-inflammatory properties both in in vitro and in vivo systems. Accumulating data shows that curcumin inhibits HSC activation by blocking leptin signaling, regulating intracellular glucose and its derivatives and modulating lipid metabolism, as well as balancing formation and degradation of ECM, in combating liver fibrogenesis.^[34]^ Demethoxycurcumin is a naturally occurring curcumin analogue, and there have been few studies on the treatment of liver diseases with demethoxycurcumin until now.^[35]^ Notably, our molecular docking results showed that both curcumin and demethoxycurcumin strongly bound to MAPK1, EGFR and SRC. For future research, more attention need to be paid to them.

In GO analysis, main components of CL were involved in positive regulation of cell migration, positive regulation of cell motility, and positive regulation of cellular component movement. All of these BP are closely associated with ECM remodeling and recruitment of inflammatory cells, which is of great importance in the progression of hepatic fibrosis. Main involved cellular components included ECM, external encapsulating structure, and vesicle lumen, and MF concerned included carbonate dehydratase activity, phosphotransferase activity, and kinase activity. KEGG pathway enrichment analysis revealed that HIF-1 signaling pathway, Phospholipase D signaling pathway, Relaxin signaling pathway, Endocrine resistance and PI3K/Akt signaling pathway were most enriched pathways associated with hepatic fibrosis. As the most significantly enriched pathway, HIF-1 signaling pathway mediates the body responses to hypoxic microenvironment, induces the angiogenesis, migration and proliferation of fibroblasts and keratinocytes, anaerobic metabolic transformation, and systemically increases the number of red blood cells. The role of HIF-1 in the development of hepatic fibrosis has been clearly identified by experiments, which closely interacts with VEGF, PI3K/Akt, MAPK, TGF-β and NF-kB signaling pathways, and plays an important role in HSC activation and ECM synthetization.^[36,37]^

A network of the common targets and pathways enriched showed that AKT1, MAPK1, PIK3CB, PIK3CA, RELA, EGFR, MTOR, SRC, CCND1, and BCL2 possibly participated in the above KEGG pathways. Considering the main targets showed by PPI network, we speculate that AKT1, MAPK1, EGFR, MTOR, and SRC may be the core potential targets of CL against hepatic fibrosis, and molecular docking was further performed. As mentioned above, both curcumin and demethoxycurcumin were strongly bound to MAPK1, EGFR, and SRC, which indicated that curcumin and demethoxycurcumin might be the core components against hepatic fibrosis, and MAPK1, EGFR, and SRC might be the core targets.

MAPK1, also known as extracellular signal-regulated kinase 2, acts as an essential component of the MAPK/ERK cascade, which mediates intracellular signaling triggered by extracellular stimuli such as growth factors and cytokines as well as by intracellular stimuli such as oxidative and endoplasmic reticulum stress and gives rise to various cellular responses including proliferation, migration, differentiation, survival or apoptosis, autophagy, and inflammatory reactions.^[38]^ MAPK cascade usually consists of at least 3 core kinases, defined as MAP3K, MAPKK, and MAPK. Once activated, the signal is propagated through sequential phosphorylation and activation of sequential kinases, which, in turn, leads to the phosphorylation of hundreds of target regulatory proteins identified in the cytoplasm, mitochondria, endoplasmic reticulum, and Golgi apparatus, as well as in the nucleus.^[39]^ Previous studies showed that, during the progression of hepatic fibrosis, MAPK1 played a crucial role in the transduction of proliferative stimuli into HSC, activation of type I collagen and Connective tissue growth factor synthesis, migration in response to chemoattractants and ROS, etc.^[3,40,41]^ Up to now, anti-fibrogenic drugs and strategies, including curcumin, have been designed to directly affect MAPK1 or to affect the pathways upstream to MAPK cascade, but few studies have reported some benefit in hepatic fibrosis patients.^[42–45]^ Based on our results, MAPK1 plays an important role in 19 of 20 most enriched pathways in KEGG analysis and strongly binds to core components of CL. It worthy to be further studied as a potential core target of CL against hepatic fibrosis.

EGFR, also known as ErbB1 or HER-1, is a transmembrane protein receptor endowed with tyrosine kinase activity.^[46]^ EGFR can bind ligands of the EGF family and activating several signaling cascades, such as the ras/raf/MEK/MAPK pathway, p38-MAPK, phospholipase C/protein kinase C pathway, the PI3K/Akt–mTOR pathway and the STAT pathway, to convert extracellular cues into appropriate cellular responses. Important evidence has accumulated on the central role of the EGFR signaling system in conveying strong reparative and regenerative signals to hepatocytes upon liver injury and inflammation.^[47,48]^ Studies also showed that different EGFR ligands exert anti-fibrogenic or pro-fibrogenic functions by activating different EGFR downstream signaling pathways.^[49,50]^ Drugs and natural components have been reported to inhibit the the phosphorylation of EGFR and its downstream pathways and prevent the progression of cirrhosis and regressed fibrosis in different animal models.^[51,52]^ EGFR was involved in 10 of 20 most enriched pathways and had effective free energy against key components in molecular docking. Further verification and exploration needs to be done in future studies.

SRC consists of 4 SRC homology domains, and is usually activated following engagement of different classes of cellular receptors, including immune response receptors, integrins and other adhesion receptors, receptor protein tyrosine kinases, G protein-coupled receptors as well as cytokine receptors. SRC participates in signaling pathways that control a diverse spectrum of biological activities including gene transcription, immune response, cell adhesion, cell cycle progression, apoptosis, migration, and transformation. Members of the SRC family kinases have been broadly investigated in cancer due to their pro-oncogenic characteristics,^[53,54]^ and results for SRC targeting have also been reported in the treatment of hepatic fibrosis.^[55,56]^

Taken together, the potential core targets showed in our study, like MAPK1, EGFR, SRC, etc, have been reported to play an important role in some significant enriched pathways, like HIF-1 signaling pathway and PI3K/Akt pathway. Potential core active components including apigenin and curcumin, have also been reported to exert an anti-fibrotic function via targets and pathways mentioned above. Our molecular docking results further verified that these components could bind closely to potential core targets. Therefore, the therapeutic effect of CL on hepatic fibrosis may be attributed to these potential core components, core targets and signaling pathways.

Our study was not without limitations. First, since the network pharmacology research was conducted based on existing database, which might be incomplete, the results were deductions based on previous experimental results and computer simulation predictions, and the potential core targets and mechanisms obtained need experimental verification in vivo or vitro. Furthermore, the binding affinity of potential core active components with potential targets awaits further verification.

In conclusion, these findings implicated that CL mainly acted into multi-component, multi-target, multi-pathway in treating hepatic fibrosis. Potential core components, core targets and signaling pathways were screened by network pharmacology analysis. Further pharmacological experiments are needed to validate the above therapeutic mechanisms of CL. Our study combined bioinformatics analysis, network pharmacology, and molecular docking to reveal the potential core components, targets and mechanisms of CL, and might provide a theoretical basis for the use of CL against hepatic fibrosis.

## Author contributions

**Conceptualization:** Qiang Han, Peng Zhang.

**Data curation:** Qiang Han, Jiahui Zhu.

**Formal analysis:** Qiang Han, Jiahui Zhu.

**Funding acquisition:** Peng Zhang.

**Methodology:** Peng Zhang.

**Software:** Qiang Han, Jiahui Zhu.

**Writing – original draft:** Qiang Han, Jiahui Zhu.

**Writing – review & editing:** Peng Zhang.
